# Prevalence and Patterns of Five Dental Anomalies in Athletes in Qatar: A Panoramic Radiographic Study

**DOI:** 10.1155/ijod/6698428

**Published:** 2026-01-19

**Authors:** Atef Hashem, Dania Almasri, Karim Chamari, Montassar Tabben, Noof AlMabrd, Mohammed Alsaey

**Affiliations:** ^1^ Sports Dentistry Department, Aspetar Orthopaedic and Sports Medicine Hospital, Doha, Qatar, aspetar.com; ^2^ Research, Naufar Center, Doha, Qatar; ^3^ Aspetar Sports Injury and Illness Prevention Program (ASPREV), Aspetar Orthopaedic and Sports Medicine Hospital, Doha, Qatar, aspetar.com

**Keywords:** hypodontia, impaction, supernumerary, taurodontism, transposition

## Abstract

**Objective:**

This study investigated the prevalence of dental anomalies within the athlete population in Qatar using panoramic radiographs.

**Design:**

This retrospective, cross‐sectional study was conducted at Aspetar Hospital in Qatar.

**Materials and Methods:**

Digital panoramic radiographs of 5000 records of athletes attending dental department were investigated for dental anomalies affecting tooth position, such as impaction and transposition; anomalies affecting tooth number, such as hypodontia and supernumerary teeth; and anomalies affecting tooth shape such as taurodontism. Panoramic radiographs were obtained over a 15 year period (March 2007 to September 2022). Radiographs were evaluated by two experienced dentists: a prosthodontist and an endodontist with a minimum of 12 years of experience. To assess interexaminer agreement, a random sample of 20 radiographs were evaluated independently by each examiner. The agreement was calculated using Cohen’s kappa statistics, with a strong level of agreement achieved (*K* > 0.95). Discrepancies were resolved by consensus discussion.

**Results:**

An initial total of 5000 athlete records from individuals attending the dental department were reviewed. After applying the study’s inclusion and exclusion criteria, 1483 records were excluded as they did not meet the specified requirements. Consequently, a final sample of 3517 athletes (mean age: 21.3 ± 7.07 years) was included in the analysis, with a predominance of males (3142; 89.3%) compared to females (375; 10.6%). Among those athletes, 1003 (28.5%) had at least one dental anomaly, and 73 athletes (2.1%) had two anomalies. The prevalence of anomalies affected teeth position, shape, and number were 20.0%, 7.7%, and 2.9%, respectively. The most observed dental anomalies were tooth impaction (19.8%), followed by taurodontism (7.7%). The prevalence of hypodontia was 1.8% and that of supernumerary teeth was 1.1%. Tooth transposition was the least found with a prevalence rate of 0.2%.

**Conclusion:**

Our findings demonstrate a notable presence of dental anomalies among athletes in Qatar, with tooth impaction being the most prevalent, followed by taurodontism and hypodontia. This highlights the importance of early detection and management of such anomalies.

## 1. Introduction

Dental anomalies arise from complex interplays between genetic, epigenetic, and environmental factors during human dental development [1]. These anomalies, either occurring in isolation or as part of a syndrome [2], are frequently observed in clinical practice [3]. While the general population shows dental anomalies prevalence ranging from 5.4% to 45% [4, 5], athletes—a group often underrepresented in these studies—might experience different impacts of these dental anomalies due to their unique lifestyle and health challenges. Understanding dental anomalies morphogenesis and its variations is crucial for health prevention and management and forms a foundational aspect of multidisciplinary clinical treatment [6]. Athletes face unique oral health challenges, including nutritional deficiencies and exercise‐driven immune suppression, often exacerbated by a lack of awareness or negative health behaviors, contributing to poor oral health [7].

In the Middle East, dental anomaly studies show a prevalence range of 18.17%−45.1% [8, 9] in the community, with specific studies in Qatar reporting prevalence for hypodontia ranging from (6.2%−7.8%) and a prevalence of (1.6%) for supernumerary teeth [10–12]. However, the prevalence of dental anomalies in Middle Eastern athletes remains unexplored. Investigating dental anomalies in athletes is crucial for enhancing their oral care, overall health, and quality of life, which in turn can optimize their training and performance capabilities [13–16].

Impacted mandibular third molars are associated with pain, swelling, and functional limitations that significantly impair patients’ quality of life prior to extraction. Moreover, the surgical removal of these teeth, although necessary, leads to a temporary decline in quality of life due to postoperative pain, restricted mouth opening, difficulty in eating and speaking, and reduced physical activity, particularly within the first week following surgery [13].

Hypodontia has been shown to have detrimental psychological consequences on young people. On the child perception questionnaire (CPQ), people with more than two missing teeth performed noticeably worse than those without hypodontia [15]. Since hypodontia requires long‐term care, it is essential for dentists to identify the condition early on, develop a treatment plan and treat it before it begins to negatively impact their psychological well‐being.

In a study that was published by Needleman et al. [16], evaluating the impact of oral health on athlete performance during the London 2012 Summer Olympics, more than 40% of athletes reported concerns related to their oral health, with 28% indicating a negative impact on their quality of life and 18% reporting adverse effects on their training and performance.

Panoramic radiographs offer a comprehensive view of the orofacial area and are a reliable diagnostic tool for developmental dental anomalies [17–19]. Therefore, to bridge the gap in our understanding of dental anomalies in athletes and their potential impact, this study endeavors to elucidate the prevalence of these conditions within the athlete population in Qatar using panoramic radiographs.

In addition to radiographic imaging, clinical examination remains a critical element in diagnosing dental anomalies, allowing for the identification of variations in tooth number, morphology, and eruption patterns through direct observation. While panoramic radiographs provide a useful two‐dimensional overview of orofacial structures, they may not fully capture the complexity of three‐dimensional anatomy. Panoramic radiography, when combined with clinical evaluation, continues to be a dependable and accessible method for initial assessment in both general practice and research settings [20].

## 2. Materials and Methods

### 2.1. Study Design and Setting

This retrospective, cross‐sectional study was conducted at Aspetar Orthopaedic and Sports Medicine Hospital in Doha, Qatar. We evaluated a cohort of athletes who visited the Dentistry Department for dental treatment.

### 2.2. Selection and Sampling

Panoramic radiographs were obtained over a 15 year period (March 2007 to September 2022). Exclusion and inclusion criteria were applied to the first 5000 records provided by the institution’s IT department in alphabetical order, which allowed us to create the final study population.

### 2.3. Inclusion Criteria

• Athletes aged 12 years and above.
• Presence of high‐quality panoramic radiographs.

### 2.4. Exclusion Criteria

• Non‐athletes.
• Absence of panoramic radiographs.
• Athletes below 12 years old.
• Poor‐quality radiographs.
• Incomplete records.
• Athletes with syndromic craniofacial anomalies.

After applying these criteria, the final sample comprised 3517 athletes (age: 21.3 ± 7.07 years), with a male predominance (3142, 89.3% males, and 375, 10.6% females).

Digital panoramic radiographs were acquired with two radiographic equipment (Orthophos XG/XG 5 DS/Ceph and Orthophos XG^Plus^ DS/Ceph; and Sirona Dental System, Long Island City, NY) using the following parameters: 64 kV, 8 mA, 14 s) over a period of 15 years (from March 2007 to September 2022, include). Both radiographic equipment were provided by the same manufacturer, and the exposure settings were standardized. Using two different radiographic equipment from the same manufacturer, set at the same imagery settings, is not expected to affect the outcomes of our study. Indeed, the anomalies investigated can be easily identified on any good quality panoramic radiograph. Radiographs were stored in a unique digital database (Dental 4 Windows SQL Vi6 Copyright 1992–2021 Centaur Software). Given the substantial volume of panoramic radiographs (*n* = 5000), the evaluation was conducted by two examiners to expedite the analysis and facilitate timely completion of the study. To assess interexaminer reliability, 20 radiographs were randomly selected and independently examined by two experienced dentists on a computer monitor 23” diagonal IPS widescreen WLED backlit anti‐glare LCD; maximum resolution of 1920 × 1080 HP EliteOne 800 G1 All‐in‐One Business PC with subdued ambient lighting.

### 2.5. Categorization of Dental Anomalies

Dental anomalies were classified into three types and five subtypes:

1. Anomalies affecting tooth position: impaction and transposition.
2. Anomalies affecting tooth number: hypodontia and supernumerary teeth.
3. Anomalies affecting tooth shape: taurodontism.

### 2.6. Tooth Impaction

Archer, quoted Mead’s (1954) definition of tooth impaction as “a tooth that is prohibited from erupting into its normal location because of malposition, deficiency of space, or other obstructions” [21].

### 2.7. Tooth Transposition

Positional interchange of two adjacent teeth—particularly by the roots—or the development or eruption of a tooth in a position normally occupied by a nonadjacent tooth [22].

### 2.8. Nonsyndromic Hypodontia

It refers to the developmental failure of teeth. Generally, hypodontia refers to the condition where there is absence of one or a few teeth. Oligodontia is used to describe six or more missing teeth, and anodontia is the complete absence of teeth. It may occur as part of a recognized genetic syndrome or as an isolated trait.

### 2.9. Supernumerary Teeth/Tooth

Supernumerary teeth or hyperdontia are defined as the existence of an excessive number of teeth in relation to the normal dental formula (20 in the deciduous dentition and 32 in the permanent dentition) [23].

### 2.10. Taurodontism

Taurodontism is a morphological alteration of the tooth resulting from the failure of Hertwig’s epithelial root sheath diaphragm to invaginate at the normal horizontal level. It is characterized by an enlarged pulp chamber, apical displacement of the pulpal floor, and the absence of constriction at the cemento‐enamel junction [24].

### 2.11. Data Recording and Ethical Considerations

Data was recorded using Microsoft Excel (Microsoft 365 Apps for enterprise) and prepared for descriptive statistical analysis. Ethical approval of the research was obtained in accordance with the ethical standards of the Helsinki Declaration from Aspire Zone Foundation Institutional Review Board (application approval number: E202111027).

## 3. Results

An initial total of 5000 athletes attending the dental department were reviewed. After applying the study’s inclusion and exclusion criteria, 1483 records were excluded as they did not meet the specified requirements. Consequently, a final sample of 3517 records were assessed, of which 3142 (89.3%) were males (age: 21.5 ± 6.97 years) and 375 (10.6%) were females (age 19.9 ± 7.74 years).

Of the 3517 athletes, 1003 (28.5%) had at least one dental anomaly, and 73 athletes (2.1%) had two anomalies. No patient had more than two simultaneous dental anomalies.

**Figure 1:**
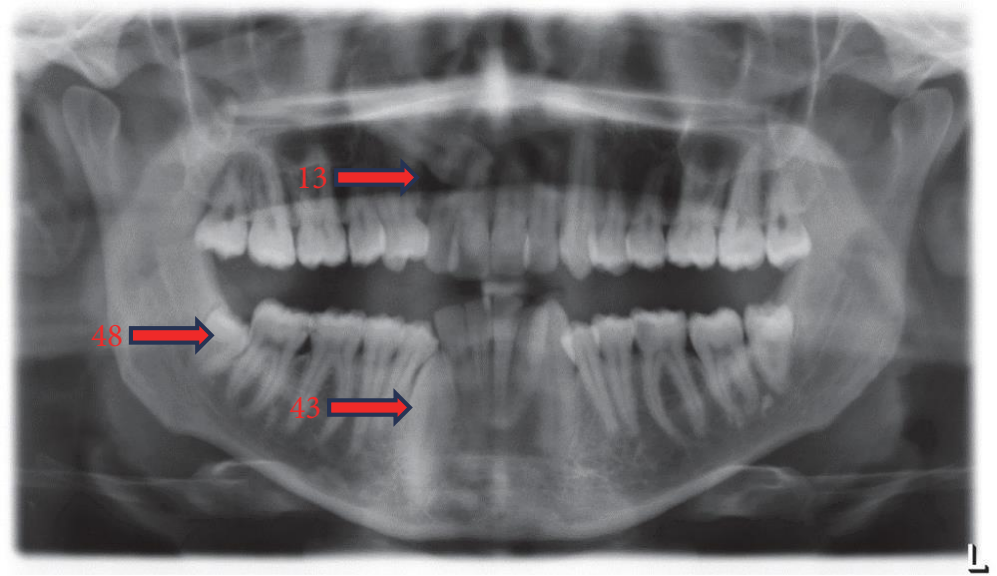
Orthopantomogram showing impacted maxillary right canine (13) and right mandibular canine (43) in addition to mandibular right third molar (48).

**Figure 2:**
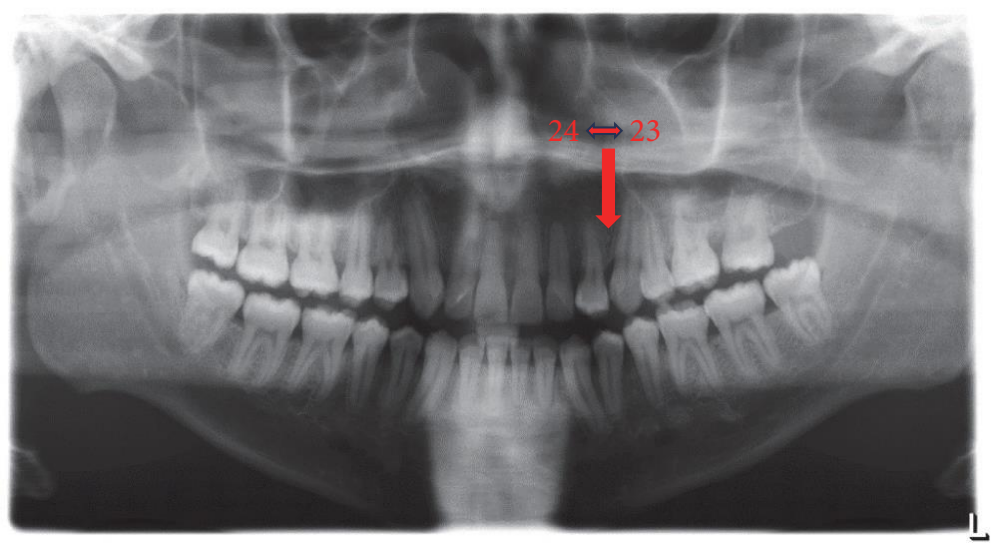
Orthopantomogram showing maxillary left canine (23) and first premolar (24) transposition.

**Figure 3:**
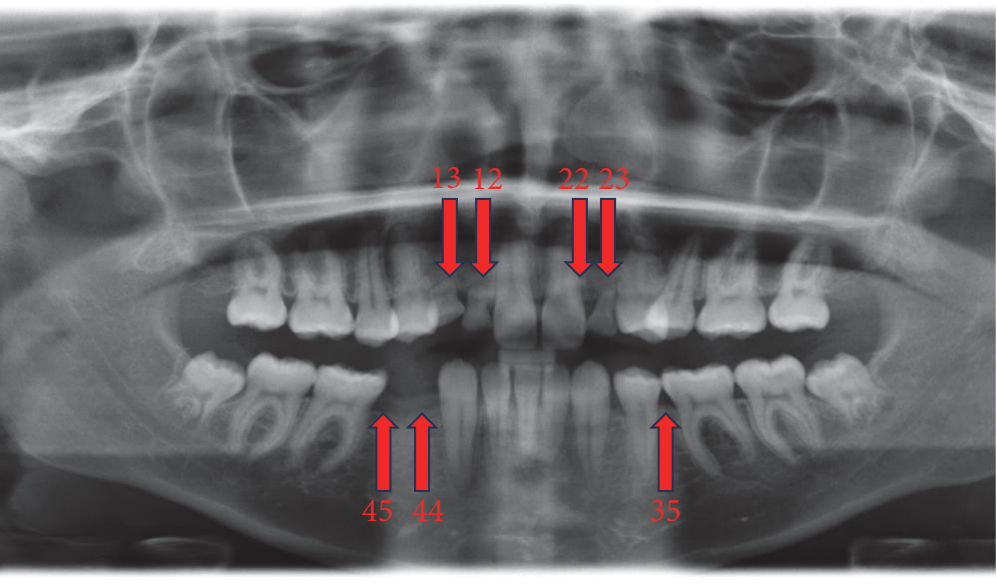
Orthopantomogram showing hypodontia of maxillary canines (13, 23), and lateral incisors (12, 22). In addition to mandibular left second premolar (35) and right first and second premolar (44, 45).

**Figure 4:**
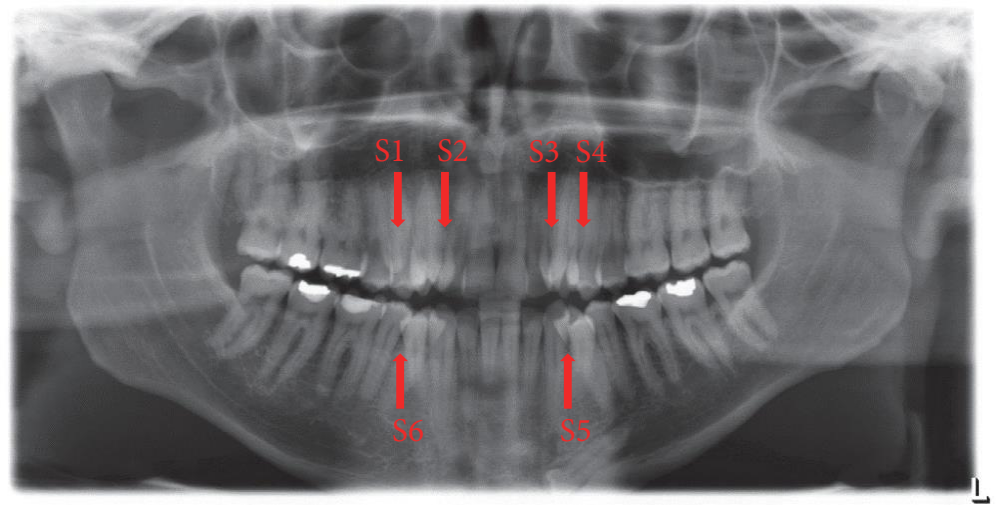
Orthopantomogram showing six supernumerary teeth in the maxillary premolars (S1, S4) and lateral incisors (S2, S3) area and mandibular premolar areas (S5, S6).

**Figure 5:**
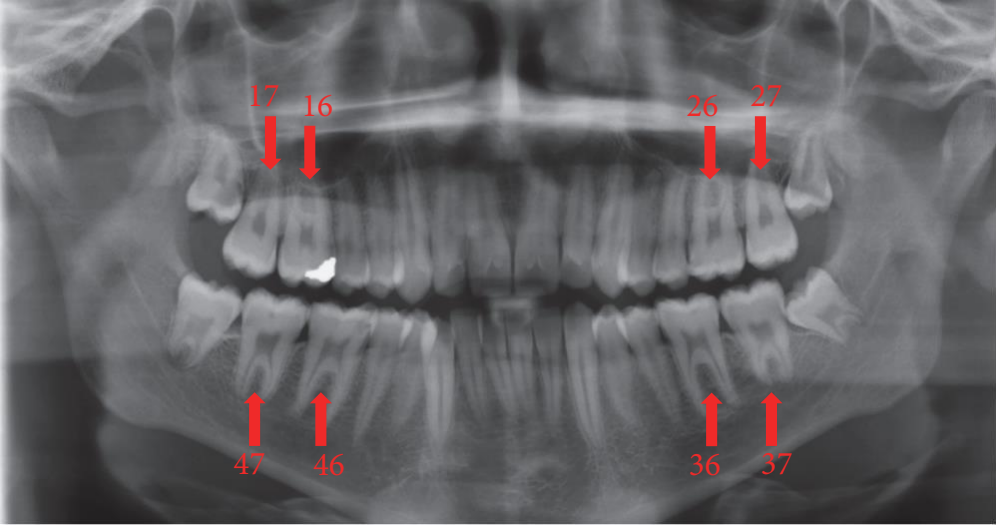
Orthopantomogram showing bilateral taurodontism affecting maxillary second and first molars (17, 16, 26, 27) and mandibular first and second molars (37, 36, 46, 47).

The prevalence of the anomalies that affected teeth positions, numbers, and shape were 20.0%, 2.9%, and 7.7%, respectively. The prevalence rates of each dental anomaly are shown in Table 1. Figures 1–5 illustrate a sample of the radiographic appearance of these anomalies.

**Table 1:** Prevalence of dental anomalies

Condition	Number of patients (out of 3517)	Prevalence (case/100 patients)
Tooth position	**701**	**20.0**
Impaction	695	19.8
Transposition	6	0.2
Tooth number	**684**	**2.9**
Hypodontia	644	1.8
Supernumerary	40	1.1
Tooth shape	**271**	**7.7**
Taurodontism	271	7.7

*Note:* Values in bold are the sum of each subcategory.

Interexaminer reliability was excellent, with a kappa coefficient exceeding 0.95. To ensure consistency between examiners, calibration was conducted using a subset of 20 randomly selected patient panoramic radiographs. The identification numbers were recorded in a data collection sheet and shared with the two examiners via e‐mail. Each examiner independently reviewed the corresponding radiographs and scored the variables. Subsequently, the results were compared, and the interexaminer agreement was calculated using the kappa statistic.

## 4. Discussion

The aim of our study was to assess the prevalence of developmental dental anomalies detectable on panoramic radiographs in registered athletes in the State of Qatar.

While numerous studies have explored the prevalence of developmental dental anomalies in diverse global populations, revealing varying rates, there remains a notable gap in research specifically focusing on athletes in the Middle East, a demographic not yet thoroughly investigated in this context.

In this cohort of 3517 athletes, developmental anomalies were common, with position anomalies predominating (20.0%), driven largely by tooth impaction (19.8%), followed by shape anomalies; taurodontism (7.7%), while number anomalies were less frequent (hypodontia 1.8% and supernumerary 1.1%).

The prevalence of impaction corresponds to the age at which third molars complete development and space limitations become clinically evident. Among athletes, demanding training schedules may delay routine dental care, resulting in later detection during opportunistic imaging. In addition, participation in contact and collision sports can increase the need for radiographic surveillance (e.g., trauma), thereby raising the likelihood of identifying impacted teeth that might otherwise remain undocumented [25]. In athletes, impacted mandibular third molars can cause pain, swelling, and temporary functional limitations following extraction, which may disrupt training and performance. Early detection of dental impactions helps minimize the risk of unplanned time loss and enables more proactive and efficient dental care strategies [26].

The presence of taurodontism may complicate various dental interventions such as extractions, endodontic procedures, prosthodontic restorations, periodontal therapy, and orthodontic treatment, underscoring its relevance in clinical dentistry [24]. In athletes, where timely care is often essential, recognizing taurodontism during routine dental check‐ups and through careful evaluation of panoramic radiographs can aid treatment planning and potentially shorten return‐to‐play timelines following dental emergencies.

Although less prevalent, number anomalies carry psychosocial and occlusal implications. In younger athletes, hypodontia can influence esthetics, occlusion, and appliance design, while supernumeraries may disrupt eruption paths or complicate trauma management [15, 23]. Timely detection during adolescent screening may facilitate preventive strategies, limit malocclusion development and simplify subsequent orthodontic care.

In our study, the total prevalence of developmental dental anomalies was 30.6% of which 1003 athletes (28.5%) had at least one dental anomaly, and 73 (2.1%) had two anomalies. This is within the range of findings of other studies in and around the middle east [18, 27, 28]. The variation is likely to be related to the nature of the population examined, variable sampling techniques and different diagnostic criteria. Whilst the age range of patients investigated in the above studies is comparable to our study with slight variations between the lower and upper age limits, all the above studies investigated the most common dental anomalies seen on panoramic radiograph like our study. However, there were variations amongst studies as additional types of rare dental anomalies were also investigated along with the most common dental anomalies. This is a common finding in dental literature and hence the total prevalence of dental anomalies in these studies, including our study, should be interpreted bearing this variation in mind.

The most observed dental anomalies were related to tooth position 20%, where impaction was the highest reported anomaly (19.8%). This is comparable to the findings of studies in the Middle East [8, 18, 29]. Afify and Zawawi [8] examined 878 patients and found that the prevalence of tooth impaction was 21.1%. Bilge et al. [18], on the other hand, analyzed the data of 1200 patients and found that 17.83% had tooth impaction. Similarly, Shayan et al. [29] investigated a younger age group population, and the prevalence of impaction was 21.8%. However, the prevalence of tooth impaction in our study was higher than those reported by other studies which reported a prevalence rate of 3.41%, 7.6%, and 6.4%, respectively [9, 30, 31]. This variation may be related to the younger age of the population investigated in those studies, as teeth which may look likely to erupt at a younger age may change their course and become impacted and vice versa.

The prevalence of tooth transposition in our study was 0.2%, which is close to the reported prevalence between 0.25% [32] and 0.66% [33] with an overall prevalence of 0.33% [22]. More recent studies showed equivalent results to our study ranging from 0.18% to 0.5% [9, 18, 27, 30]. This may indicate that the prevalence of tooth transposition does not tend to dramatically change between different ages or ethnic groups, nor does it seem to be markedly different in the athletes of our study.

Tooth shape anomaly such as taurodontism was the second most common anomaly with a prevalence rate of 7.7%. This is slightly lower than the average prevalence of 11.8% reported by Decaup et al. [34] in their meta‐analysis. However, other studies reported wide variations in the prevalence rate of taurodontism ranging from 0.1% in Saudi Arabia to 22.9% in Iran [27, 35]. The variations in taurodontism prevalence between different studies was attributed to the high heterogeneity across the studies evaluated [34, 36], this is likely due to differences in sample sizes, research methodology and the nature of the population investigated.

Anomalies related to tooth numbering were the least present with a prevalence of 1.8% for hypodontia excluding third molars and 1.1% for supernumerary teeth. Our observed hypodontia prevalence was lower than the general prevalence of 6.4% reported by Khalaf et al. [37]. Comparatively, studies in Qatar focusing on a younger demographic noted hypodontia rates of 6.2% and 7.8% [10, 11]. This discrepancy in our study might stem from our specific age group, where only athletes with retained primary teeth were identified as hypodontia cases, thereby reducing potential overestimation due to tooth extractions. As for supernumerary teeth, our finding of 1.1% aligns well within the broader reported range of 0.5%–5.3% [38, 39], indicating a consistency with global prevalence trends.

## 5. Study Limitations

The study’s findings may be impacted by several factors. First, there is a sample selection bias as it only included athletes, who visited the dentistry department, potentially overlooking individuals without dental concerns or access to care. Second, the age group representation skews towards those over 12 years old, potentially misrepresenting the prevalence of dental anomalies in younger athletes. Third, the cohort was clinic‐attending and predominantly male, and because anomaly ascertainment relied on panoramic radiographs rather than standardized clinical‐radiographic protocols, selection and information biases likely influenced absolute prevalence estimates and may limit generalizability; these factors also constrain the interpretability of subgroup comparisons. Additionally, being retrospective, the study relied on existing records, introducing potential limitations in data completeness and quality. Finally, the geographical limitation to Qatar may restrict the generalizability of the findings to other regions or populations, indicating a need for caution in extrapolating the results beyond this context.

## 6. Conclusion

Our findings demonstrate a notable presence of dental anomalies among Qatari athletes, with tooth impaction being the most prevalent, followed by taurodontism and hypodontia. These insights are vital for dental practitioners, highlighting the importance of early detection and management of such anomalies.

## Conflicts of Interest

The authors declare no conflicts of interest.

## Funding

This research did not receive any specific grant from funding agencies in the public, commercial, or not‐for‐profit sectors.

## Data Availability

The data that support the findings of this study are available from the corresponding author upon reasonable request.
